# Senescence and Cancer: A Review of Clinical Implications of Senescence and Senotherapies

**DOI:** 10.3390/cancers12082134

**Published:** 2020-07-31

**Authors:** Lynda Wyld, Ilaria Bellantuono, Tamara Tchkonia, Jenna Morgan, Olivia Turner, Fiona Foss, Jayan George, Sarah Danson, James L. Kirkland

**Affiliations:** 1 The Healthy Lifespan Institute, Department of Oncology and Metabolism, The Medical School, University of Sheffield, Beech Hill Road, Sheffield S10 2RX, UK; i.bellantuono@sheffield.ac.uk (I.B.); j.morgan@sheffield.ac.uk (J.M.); olivia.turner@aptusclinical.com (O.T.); jayan.george@aol.com (J.G.); s.danson@sheffield.ac.uk (S.D.); 2 Robert and Arlene Kogod Center on Aging, Mayo Clinic, Rochester, MN 55905, USA; Tchkonia.Tamar@mayo.edu; 3 Department of Pathology, Sheffield Teaching Hospitals NHS Foundation Trust, Sheffield S10 2JF, UK; fiona.foss@nhs.net; 4 Departments of Internal Medicine, Geriatric Medicine and Gerontology, The Mayo Clinic, Rochester, MN 55905, USA; Kirkland.James@mayo.edu

**Keywords:** senescence, cancer, aging, frailty, senolytics, senotherapies, geriatric oncology

## Abstract

Cellular senescence is a key component of human aging that can be induced by a range of stimuli, including DNA damage, cellular stress, telomere shortening, and the activation of oncogenes. Senescence is generally regarded as a tumour suppressive process, both by preventing cancer cell proliferation and suppressing malignant progression from pre-malignant to malignant disease. It may also be a key effector mechanism of many types of anticancer therapies, such as chemotherapy, radiotherapy, and endocrine therapies, both directly and via bioactive molecules released by senescent cells that may stimulate an immune response. However, senescence may contribute to reduced patient resilience to cancer therapies and may provide a pathway for disease recurrence after cancer therapy. A new group of drugs, senotherapies, (drugs which interact with senescent cells to interfere with their pro-aging impacts by either selectively destroying senescent cells (senolytic drugs) or inhibiting their function (senostatic drugs)) are under active investigation to determine whether they can enhance the efficacy of cancer therapies and improve resilience to cancer treatments. Senolytic drugs include quercetin, navitoclax, and fisetin and preclinical and early phase clinical data are emerging of their potential role in cancer treatments, although none are yet in routine use clinically. This article provides a review of these issues.

## 1. An Aging Population and Cancer

In the western world, life-expectancy has doubled in the past 200 years and, whilst most of the early gains were in childhood longevity, in the past 70 years the majority of gains have been in older people [1,2]. Consequently, the global proportion of people over 65 is predicted to increase from 18% now to 26% in 2041 [3]. The oldest old group of over 85 year olds is also predicted to double over the same period, from 2 to 4% [3]. This increase in lifespan has been accompanied by an increase in rates of chronic disease. Consequently, life-span has outpaced health-span [4], as the burden of chronic health conditions has increased in this older population. As a result almost 50% of over 75s have 2 or more chronic health conditions [3].

One such age-related condition is cancer. Cancer is more common in older age groups with predictions of substantial increases in the next 15 years of approximately 50% in developed countries, (largely due to the increase in the older population), and even higher in developing and middle income countries [5]. Aging interacts with cancer in a range of ways: some of the molecular pathways and causes of aging [6] and the pathways and causes of cancer [7] overlap significantly. Aging is integral to the causation of many cancers and also impacts on treatment response, prognosis, treatment allocation, and treatment tolerance. One of the key processes of aging—senescence—links aging and cancer together, and this is the focus of this article.

## 2. Biological Changes of Aging and Senescence

Aging is a complex process involving multiple factors, including genetic and epigenetic alterations to DNA, cellular damage from reactive oxygen species (ROS) produced by incomplete aerobic metabolism, age-dependent decline in mitochondrial function, stem cell exhaustion, and telomere shortening [6,8]. The rate at which aging occurs is highly variable and depends on both polygenic hereditary factors [9] and a wide range of environmental factors (such as obesity, diet, exercise, and exposure to drugs and chemicals), which is why biological and chronological age are often mis-matched. A key factor in the aging process is cellular senescence. Senescent cells are rare in the tissues of young organisms but become more common as tissues age, especially in adipose tissue, muscle, and skin [10].

At a cellular level senescence refers specifically to a progression of complex changes culminating in the loss of proliferative potential (which may be reversed in some circumstances), the inhibition of cellular apoptosis, chromatin alterations, and metabolic and synthetic changes. Senescent cells release a complex, cell type-specific, mixture of bioactive molecules (senescence-associated secretory phenotype or SASP), which impacts on the adjacent cells and extracellular matrix and contributes to age-related tissue degeneration.

Senescence is increasingly recognized as a heterogeneous group of cell states, depending on the type, degree, and duration of stimuli that triggered it [11]. Senescence may also be categorized as acute or chronic, depending on the size and duration of the stimulus. Acute senescence is a short-term process, often triggered to limit an inflammatory response or physiological process, such as wound healing or embryological development [12]. Chronic senescence is triggered by feedback loops when the senescent stimulus is prolonged, and may cause diseases such as atherosclerosis, arthritis, neurodegenerative disease, and diabetes [13] as well as effecting classical age-related decline in tissue function [14].

There are several regulatory pathways through which senescence may be stimulated under appropriate conditions. These include the pathways for cellular proliferation (the cell cycle and its regulatory machinery), the regulation of apoptosis, the DNA damage response (DDR) pathway, cellular energy metabolism, and the unfolded protein response (UPR) [15]. In turn, senescence may impact on these same pathways via positive or negative feedback loops as well as other intracellular pathways and extracellular processes, such as the immune modulation, inflammation [16], regulation, and maintenance of the extracellular matrix, and angiogenesis [17]. Consequently, senescence can have wide ranging impacts on tissue structure and function.

**Figure 1:**
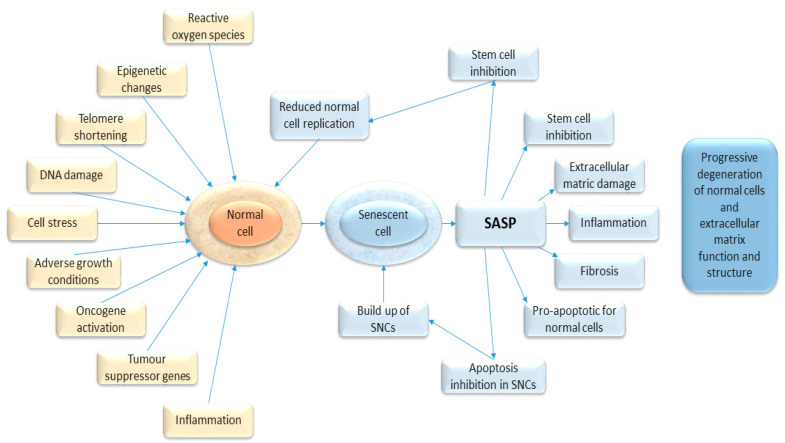
Diagram showing the causal stimuli which may trigger senescence (in yellow) and the effects of senescence on the host tissue (in blue).

Many of these pathways are also involved in the process of cancer development, suppression, progression, recurrence, and response to therapies (Figure 1). The interactions of senescence and cancer are therefore complex and poorly understood at present.

It has been proposed that, whilst senescence itself is largely tumour suppressive [18], the SASP of chronically senescent cells may promote tumour recurrence or progression [19]. There are also potential interactions with cancer therapies, in particular, radiotherapy and chemotherapy, which may trigger either cell death at high doses or senescence at non-lethal doses. This induced senescence may be viewed as a cytostatic clinical response, but some of these therapy induced senescent cells may reactivate and lead to recurrence [20,21,22]. They may also be an opportunity for tumour targeting to enhance cell killing.

In addition, senescent cells, by contributing to the pathogenesis of the aging phenotype (frailty, sarcopenia, lack of resilience, and aging-related disease) [14], impact on the ability of a patient with cancer to tolerate a range of cancer therapies. Consequently, there is great academic interest in senescence, and researchers have developed reliable methods to identify these cells in tissue sections [23]. No one marker alone is sufficient to identify senescence and most researchers use a panel of biomarkers, such as senescence-associated β-galactosidase, p21, and p16^INK4A^, as well as a number of other biomarkers, such as heterochromatin and proliferation markers (Table 1) combined [24] with a marker of cellular proliferation (Ki-67, for instance).

Interest is also growing in a new group of drugs called senotherapies. These are drugs that either suppress the process of senescence or destroy senescent cells, and which may have potential in cancer therapy.

## 3. Regulatory Pathway of Senescence

The regulation of senescence is complex and incompletely understood, and a detailed review is beyond the scope of this article, but excellent reviews are available [13,25]. A range of factors may trigger senescence, including cell stressors, such as reactive oxygen species, DNA damage due to aging, radiation, and genotoxic substances (including many chemotherapy agents), age-related telomere shortening, adverse growth conditions, tumour suppressor gene activation, and oncogene activity. (These are summarized in Figure 1). Several key pathways into senescence have been described: replicative senescence, oncogene-induced senescence, stress-induced senescence, and therapy-induced senescence.

### 3.1. Replicative Senescence

The process of senescence activation is closely linked to cell cycle regulatory machinery. The Gap One (G1) phase may progress to the synthetic (S) phase or be side tracked into a reversible state of dormancy (G0), terminal differentiation, or senescence (usually, but not always, causing irreversible cell cycle arrest) depending on the activation of a range of cell cycle regulatory proteins. Key triggers for the activation of this G1-S phase checkpoint are telomere shortening and DNA damage.

Cells have a finite ability to replicate (the Hayflick Limit [26]) and once this limit is reached, they lose the ability to divide but remain alive and metabolically active as senescent cells. One of the key effectors of this transition is telomere shortening. Telomeres are tandem repeats of a TTAGGG nucleotide found at the end of all chromosomes that are progressively lost with each cell division. Once the remaining telomere is critically shortened, it triggers a DNA damage response (DDR), which induces proliferative arrests via the p53/cyclin E/CDK2/RB pathway that controls the cell cycle G1 to S phase checkpoint. As telomere shortening is irreversible in normal cells and cannot be repaired, it triggers senescence rather than temporary proliferative arrest.

The same pathway is triggered by non-lethal DNA damage, which again activates the DDR. DNA damage accumulates progressively with age but also occurs in the majority of cancers, where a key initiation event is often the loss of function of critical DNA repair pathways via loss of function of tumour suppressor genes. For example, the p53 and BRCA genes are key tumour suppressor genes that are mutated and lose function at an early stage in the process of cancer development. Again, if this DNA damage cannot be repaired, the cell may die (usually via apoptosis) or enter senescence via the p53/cyclin E/CDK2/RB signaling pathway. Whether a cell undergoes apoptosis or senescence depends on the degree of damage, and whether it is repairable. In this context, senescence is tumour suppressive in that it prevents the damaged cell from proliferating. This pathway to senescence is a key effector of chemotherapy and radiotherapy damage.

### 3.2. Oncogene-Induced Senescence

Another regulatory pathway for senescence activation is via the p16 inhibitor of kinase 4^(INK4)^, a regulatory protein that inhibits CDK4/6 and cyclin 2 and so blocks cell cycle progression via the RB protein. This pathway may be stimulated by the loss of tumour suppressor function, oncogene activation, epigenetic changes (DNA methylation, for example, which may alter gene expression), or damage to the microtubules forming the mitotic spindles (a mechanism of action of some chemotherapy agents, such as vinca alkaloids and taxanes). Once again, mild stimulation induces senescence whereas severe stimulation induces cell death.

### 3.3. Stress-Induced Senescence

Some oncogenes, such as the BRAF oncogene (which is commonly mutated in melanoma), may trigger senescence acting via mitochondrial pyruvate kinase metabolism to regulate cell cycle progression. Metabolic stress may also induce senescence and trigger the unfolded protein response (UPR). This pathway, which is designed to protect the cell from exporting misfolded proteins due to adverse environmental conditions, is activated by a range of cell stressors, such as hypoxia, acidosis, or other metabolic stresses. It triggers cell cycle arrest and senescence if the stressor is prolonged. The UPR is often activated in many cancers [27] due to their abnormal microcirculation (which leads to cellular hypoxia and acidosis) and the high glucose needs of tumours (Warberg effect), resulting in metabolic stress.

## 4. Senescence-Associated Secretory Phenotype, SASP

Whereas acute senescence is largely beneficial in the regulation of transient injury or stress, if a cell experiences prolonged non-lethal stress, a chronic senescent state is entered where the continued production of SASP perpetuates cellular and extracellular matrix damage and results in the acquisition of an aging or disease linked phenotype.

Senescent cells release SASP factors that damage nearby non-senescent cells and the extracellular matrix, triggering inflammation, fibrosis, and apoptosis of adjacent healthy cells, but the senescent cells themselves are resistant to these effects and accumulate, thus generating more SASP factors. The precise content of the SASP released by a senescent cell may vary according to the host tissue and the degree and type of senescent stimulus. In some cases, the SASP may have a beneficial effect—when triggered acutely in wound healing, for example, the SASP is involved in triggering inflammation and an immune response and, once wound closure has occurred, the myofibroblasts become senescent to prevent excessive fibrosis. Similarly, during embryogenesis senescence is a mechanism whereby certain processes may be physiologically stopped [12].

The SASP is a melange of over 100 molecules, including inflammatory cytokines, growth factors, and proteases. These include interleukins 1 and 6 (pro-inflammatory cytokines that may trigger or promote inflammation), interleukin 8 (which attracts neutrophils towards a tissue and stimulates their activity in the inflammatory process), and matrix metalloproteinases (which break down the various components of the extracellular matrix, such as collagen). The SASP of a particular senescent cell type may have a tumour suppressive function and trigger immune activation or promote tumour growth and inflammation depending on the host tissue. This variation in function makes study complex, as senescent cells in different tissues secrete slightly varying mixtures of SASP factors and so the inhibition of senescent cells may have positive or negative effects on cancer growth.

## 5. Role of Senescence in the Development of Cancer

Senescence is a key mechanism of tumour suppression. This may be mediated by the DNA damage response or via key oncogenes. Several key oncogenes, including ras, cyclin E, raf, and E2F3 expression, are linked to senescence induction and may have a tumour suppressive role [28,29,30]. This may be via the inhibition of proliferation of malignant cells or by stimulating immune surveillance. Studies have demonstrated high levels of senescence in premalignant lesions and lower levels in invasive disease, suggesting a role for senescence in blocking malignant progression [2,31,32]. Mutations in key oncogenes often trigger senescence to eliminate premalignant cells before they acquire further mutations and become invasive [33]. There is evidence of senescence being triggered by the ras oncogene during lung and pancreatic tumorigenesis [2]. Some mechanisms to evade senescence are therefore a key feature of malignant progression from pre-malignant to invasive disease. It has been proposed that a loss of one of the key senescence effectors, such as the tumour suppressors p16^INKA4^ or p53, may be mechanisms whereby the failure of senescence occurs [2]. This allows the oncogene to stimulate progression without check. Senescent cells may evoke an anti-tumour immune response—so called “senescence surveillance”—mediated by the cytokines within SASP. This has been shown to suppress the progression of malignancy in hepatocytes in a mouse model [34]. Other mechanisms of tumour suppression have been proposed [35].

In contrast there is also evidence that stromal cell senescence may have a tumour promoting effect in some models—this may be because of the proangiogenic influence of some components of SASP, such as vascular endothelial growth factor (VEGF) or the impact of senescent fibroblasts on adjacent tumour cells [17,36]. There is also evidence that “immune senescence”, associated with the aging of the immune system in older age may contribute to the failure of immune surveillance and contribute to cancer development in older people. This was shown in a study where telomere shortening in peripheral blood T cells, was linked to the development of cancer [37].

## 6. Role of Senescence as a Prognostic Marker for Cancer

Senescent cells are more common in the normal tissues of aged individuals, especially in certain tissue types, such as skin and adipose tissue [38,39,40]. They are also present in cancer and in premalignant lesions and have been evaluated as prognostic markers [41]. The detection of senescence is complicated, as many of the molecules involved in senescence signalling are also oncogenes or tumour suppressors that may be up or down regulated as part of the carcinogenic process. The assessment of combinations of markers is therefore required in addition to a marker of proliferation, such as Ki67. Common markers of senescence include β-galactosidase activity, p16^INK4A^, p21, and heterochromatin levels combined with a marker of proliferative arrest [23].

Markers of senescence have been assessed in a number of human cancers. In breast cancer, p16 levels correlate with breast cancer subtype, proliferative status, and prognosis [42]. Another study of breast cancer demonstrated that the increased expressions of senescence markers p14^ARF^ and p16^INK4a^ were associated with increased risk of disease recurrence and poor survival outcomes [43]. Another study of a range of cancer types, including breast, lymphoma, colon, sarcoma and lung, found that senescence markers were linked to better prognosis [29]. Colon and endometrial cancers have also been studied, as well as senescence markers linked to improved prognosis [44,45]. Table 2 shows a summary of a range of studies linking senescence with tumour prognosis.

## 7. Role of Senescence in Cancer Treatment Response

### Chemotherapy

**Figure 2:**
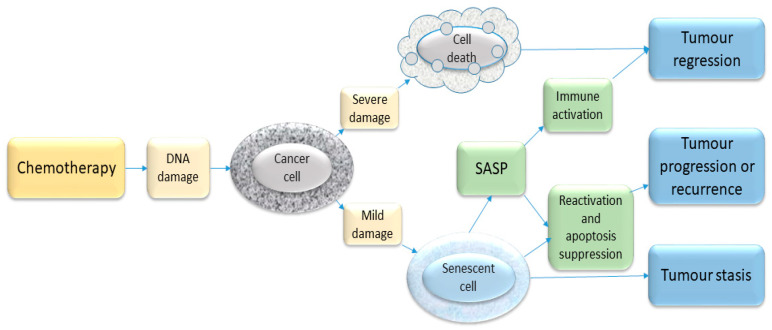
Schematic illustration of some chemotherapy-triggered responses in cancer cells. Most chemotherapeutic drugs induce DNA damage and activate the DDR. If the extent of damage is severe, the cell may die—e.g., through apoptosis. If the damage is sub-lethal, the cell may enter a state of senescence. This senescence response may represent a mechanism of inducing clinical tumour stasis (growth arrest) but in some situations such growth-arrested cells may re-enter the cell cycle and cause disease recurrence. In addition, the release of SASP by senescent cells may contribute to tumour recurrence as well as having an immune stimulatory function. The balance of these various processes will vary, depending on the host tissue and the type and degree of stimuli.

Chemotherapy may cause cell death, often by apoptosis, resulting clinically in tumour regression. It may also cause cellular senescence, leading clinically to tumour stasis (growth arrest) (Figure 2). Many types of chemotherapy cause DNA damage (DNA strand breaks or cross linking), which can, if severe, cause cell death via the DNA damage response, or they may trigger a non-lethal DDR, leading to acute or chronic senescence, depending on the extent and duration of the stimulus [52]. Entry into senescence or cell death may also depend on whether the cell has functional tumour suppressor genes, such as p53 or p16^INK4A^ to regulate cell behaviour [53]. Moderate chemotherapy doses are more likely to cause senescence and higher doses more likely to cause cell death [54]. Different types of chemotherapy damage DNA in a number of different ways. For example, doxorubicin prevents the resealing of the DNA double helix by inhibiting topoisomerase 2, which triggers a DDR and thereby may cause senescence [54]. Others, such as vinca alkaloids and taxanes, work by causing damage to the mitotic spindle during mitosis, resulting in cell death. Cyclophosphamide causes DNA cross linking, which again may trigger a DDR.

The role of senescence in response to chemotherapy is more complicated, however, in that the SASP of senescent cells induced by treatment varies between tissues and cell types, according to the precise senescent stimulus [55]. In particular, some senescent cells secrete exosomes [56] (small “packages” containing a variety of proteins and mRNA) and these may have a tumour promoter function [55]. Consequently, senescence induced by some cancer therapies may be harmful and promote tumour growth.

In a mouse model, lymphomas induced by the myc oncogene responded to cyclophosphamide by undergoing senescence mediated by p16^INK4A^ and p53 [57]. It has been hypothesised that the induction of permanent senescent cytostasis may be an effective strategy for cancer treatment, rather than killing the cancer cell [58], and may have reduced toxicity. However, there are concerns that these senescent cells may serve as a potential reservoir for resistance if their senescent state is reversed.

There are some human data about the role of senescence in chemotherapy response in lung, breast, and prostate cancer and lymphoma. In breast cancer cases treated with neoadjuvant chemotherapy, 41% of tumours were stained by senescence markers compared to only 10% of untreated cases, showing that senescence is induced by CAF (cyclophosphamide, Adriamycin, and fluorouracil) chemotherapy [53]. Senescence is also an important mechanism of efficacy in the treatment of breast cancer with PARP inhibitors [59] and many typical chemotherapy agents used to treat breast cancer, such as epirubicin and cyclophosphamide [60]. The treatment of breast cancer cells with Adriamycin for 5 days induces 60% of cells to become senescent [61]. A very small study of neoadjuvant chemotherapy in patients with non-small cell lung cancer showed increased senescence biomarkers in patients treated with a taxane and carboplatin [62]. Similarly, in prostate cancers treated with neoadjuvant mitozantrone, elevated levels of senescence biomarkers were found [31,63]. Similarly, senescence has been seen in chemotherapy-treated lymphoma [64].

Whilst cells that undergo apoptosis are permanently removed from a cancer, senescent cells remain and secrete various inflammatory cytokines, which may have both positive and negative impacts [65]. There have been concerns that these senescent cells may resist further chemotherapy damage and be a potential reservoir for recurrence. There is evidence that senescent cells may also be re-programmed to re-enter the cell cycle after certain types of chemotherapy [20,21,62] and may acquire a more “stem cell”-like phenotype, which may in turn contribute to tumour regrowth and evolution [66,67]. Recently, another form of tumour dormancy has been described whereby multinucleate senescent giant cells may re-enter the cell cycle and produce viable diploid daughter cells which may repopulate a tumour [68,69].

Conversely, there is evidence that the secretion of SASP factors may enhance the immune surveillance of tumours and induce bystander cells to become senescent thereby inhibiting tumour progression [66]. It can be seen from the above that the role of senescence in the response of cancer to chemotherapy is highly complex and more research is needed to clarify these interactions.

## 8. Role of Senescence in Radiotherapy

Radiotherapy, which is one of the mainstays of cancer therapy, acts by causing direct DNA damage and has wide ranging impacts on cancer cells mediated by reactive oxygen species. The DNA damage response is triggered and if repair is not possible, cells either die if the damage is severe or enter senescence if less severe. In irradiated cancer cells, the percentage of senescent cells therefore increases in the remaining “radio-resistant” clones [70]. The concern is that these senescent cells may be released from senescence and assume a more “stem cell”-like phenotype, which may result in aggressive recurrence [71]. Cerebral glioblastoma, in which radiotherapy induces senescent multinucleate giant cells to form and reactivate these cells, is now recognized as an important mechanism of relapse [72].

In contrast, radiotherapy also triggers an immune response, making the treated cells more immunogenic in a variety of ways [73]. Part of this immunogenicity may be due to the release of SASP factors from senescent cells.

Another way in which senescence may be a clinically important part of radiotherapy response is in causing radiation-induced fibrosis. This can be a potentially severe complication of radiotherapy, especially in the lung where pulmonary fibrosis may occur [74]. Senescent cells also appear to be linked to skin fibrosis and ulceration following radiotherapy [75]. Senolytic agents may have a role in abrogating this fibrotic response, although to date there have been no human trials to evaluate this.

## 9. Role of Senescence in Response to Hormonal Therapies (Anti-Oestrogens, Anti-Androgens)

The mechanism of action of antioestrogen therapies in breast cancer and the mechanisms of resistance are very complex, but senescence may be one of the pathways activated when disease responds to antioestrogens [76]. Similarly androgen deprivation therapy induces senescence in prostate cancer cells and the SASP has been implicated in disease progression [77].

## 10. Role of Senescence in Surgery

Surgery is the single most important curative modality in the treatment of most human solid tumours, and cure is unlikely without it for most cancer types. Successful surgery, however, requires wound healing for recovery. Wound healing is a complex multistep process and senescence may be involved in both positive and negative ways.

After an initial haemostatic phase, an inflammatory response is triggered, and a range of cells migrate into the area or differentiate locally to effect healing. Collagen synthesis and remodelling occur, and the skin re-epithelializes. A wide range of cells, biologically active molecules, and signalling systems that both start and stop the process are involved. At an early stage in the process, senescent epithelial cells and fibroblasts appear and induce myofibroblast differentiation by secretion of platelet derived growth factor AA (PDGF-AA), which is a component of the SASP [78]. These myofibroblasts assist healing by the secretion of collagen and, by their contractile properties, help to draw the wound together (contraction). In addition, senescence is involved in “switching off” the healing process to prevent excess fibrosis. Matrix metalloproteinases within the SASP help to degrade collagen as part of remodelling [79]. Consequently, the failure of senescence may have a detrimental effect on wound healing, as seen in a mouse model where senescent cells were eliminated, resulting in disorganised collagen remodelling [78].

Conversely, chronic senescence has been causally implicated in non-healing ulcers [75] and delayed wound healing in diabetes, for example [80]. Levels of senescent fibroblasts are correlated with the length of time taken by venous ulcers to heal [81]. Wound healing is impaired in older organisms [82] for a variety of reasons and it is known that the skin is an area where senescent cells accumulate with age.

Few studies have directly assessed the relationship with senescence and surgical outcomes; however, p16 expression is negatively correlated with liver regeneration after hepatectomy in older patients [83] and the molecular aging marker CDKN2A has been shown to predict renal allograft function after 1 year in renal transplant patients [84].

It is therefore not clear what impact senolytic therapies might have on wound healing in the acute surgical setting and this requires urgent further study if these agents are to be adopted into clinical practice. This is especially important in this era of multimodal therapy regimes. If senotherapies are used as adjuncts to chemotherapy, it would be critical to ensure any negative impacts on wound healing are resolved before surgery. Similarly, if senotherapies are used to enhance resilience to surgery, the acute effect on wound healing must be properly evaluated. There are currently no studies published of senotherapies on acute wound healing.

## 11. Frailty and Senescence Reducing Resilience to Cancer Therapies

Older patients are less resilient to many cancer therapies. This is due, in part, to comorbidity rates being higher in older people, but also due to age-related organ dysfunction and frailty. Frailty is present in approximately 25% of the older surgical population and is linked to longer hospital stays and higher post-surgical mortality rates [85,86]. Adverse events are also more likely following chemotherapy [87]. Biomarkers of frailty are generally linked to the presence of chronic low grade inflammation [88] and a range of inflammatory markers have been studied and correlate with poor treatment outcomes across a range of diseases [89]. It is thought that SASP factors may contribute to this age and frailty-linked inflammation, and its abrogation may alleviate it [14].

This principle was elegantly demonstrated in a mouse model, where the implantation of senescent cells into young mice induced physical dysfunction, which spread beyond the injected senescent cells and reduced lifespan. In contrast, treating naturally aged mice or senescent cell injected mice with the senolytic drug combination of dasatinib and quercetin both reduced the number of senescent cells and caused a reduction in physical dysfunction and extended lifespan [14].

In the context of chemotherapy tolerance, there is evidence that some of the adverse effects of chemotherapy are mediated by the therapy-induced senescent cells which have a pro-inflammatory effects (due to SASP) in a doxorubicin or paclitaxel treated mouse model [67]. Removal of these therapy-induced senescent cells abrogated many of the adverse effects of chemotherapy (reduced fatigue, increased activity levels, reduced cardiac functional impairment) [67]. In a separate study, again in a mouse model, the elimination of senescent cells by the use of dasatinib and quercetin, reduced the impact of radiotherapy, improved cardiac function and exercise tolerance and increased life expectancy [90]. Data in humans are also available that show that higher levels of senescence biomarkers are linked with higher rates of treatment-induced adverse events following doxorubicin chemotherapy [67].

## 12. Senotherapies

This term refers to a group of pharmacological agents that interact with senescent cells to interfere with their pro-aging impacts. There are two main categories: senolytic drugs, which selectively destroy senescent cells and senostatic drugs, which inhibit their function by suppression of their release of SASP factors. Of the two drug groups, senolytics have been more extensively studied and show promise of therapeutic value. These are of particular interest as an adjunct to chemotherapy, where the senolytic drug may be able to target cells induced to become senescent by the cancer. They may also improve treatment resilience. There are several agents under investigation.

### 12.1. Navitoclax

Navitoclax interacts with the BCL-2 pathway and prevents it from inhibiting apoptosis, causing the senescent cells to undergo apoptosis [91]. The rationale is that the chemotherapy drug causes cells to undergo either apoptosis or senescence and the Navitoclax then induces the senescent cells to undergo apoptosis by inhibiting BCL-2 [92]. This should therefore enhance cell killing.

There is widespread evidence that Navitoclax potentiates the effect of a range of anticancer therapies in vitro and in vivo (Table 3), but efficacy in clinical studies has been limited by toxicity, in particular thrombocytopenia, which significantly limits its dose in human studies. Response rates for both single agent and combination studies have been disappointing (Table 3). There have been recent attempts to increase the specificity of Navitoclax to senescent cells by combining the drug with galactose. As senescent cells have higher levels of the galactosidase enzyme (SA β-galactosidase), the release of active Navitoclax is targeted to the senescent cells, potentially reducing platelet toxicity [93]. There are currently a few early phase clinical trials in progress, but unless the toxicity issue can be addressed it may not have a role in routine oncology practice.

### 12.2. Dasatinib Plus Quercetin

The combination of dasatinib (a tyrosine kinase inhibitor) plus quercetin (a flavonoid) (D + Q) may act in part via senescence induction, although this combination has quite wide-ranging cellular impacts. It reduces levels of senescent cells in a range of in vitro models and in mouse in vivo models [14,90]. That D + Q acts principally by senolytic effects for particular conditions, as opposed to effects on these other pathways, has been indicated in studies of, for example, osteoporosis. In mice with age-related osteoporosis, D + Q is as effective in restoring bone if administered once every few weeks as opposed to continuously despite D + Q having an elimination half-life of less than 11 h. Senescent cells take weeks to reappear. Thus, it seems very unlikely that in osteoporosis the beneficial effects of D + Q are due to mechanisms requiring continuous engagement of a classical biochemical target, such as occupancy of a receptor or inhibiting an enzyme, and senolytic “hit and run” effects are more plausible [102]. Consistent with this, in the case of frailty, which can be induced in younger healthy mice by transplanting senescent cells, a brief course of D + Q eliminates senescent cells and causes long term resolution of frailty [14]. Thus, the development of successful senolytics has more in common with developing antibiotics than the old fashioned one target-one drug-one disease drug development paradigm [103,104].

While D + Q has been shown to reduce levels of senescent cells in a range of in vitro models and in mouse in vivo models [14,90], to date there are few clinical trial data of its efficacy in the cancer setting, although some evidence in non-cancer settings. For example in pulmonary fibrosis, where it is thought that the secretome of senescent fibroblasts mediates fibrosis, Quercetin and Dasatinib reduce this effect in vivo [105]. In a cancer setting, it has been used to reduce radiation-induced skin ulceration in a mouse model by reducing senescent cells in the skin [75]. In human subjects, senescent cells may be implicated in the development of radiation-induced skin ulceration as shown recently by a study where p16 expression was associated with radiation-induced ulcers in human subjects [75]. This is potentially of great interest as radiation induced fibrosis and ulceration are a major cause of morbidity following radiotherapy in clinical practice.

### 12.3. Fisetin

Flavonoids are a group of naturally occurring chemicals found widely in fruit and vegetables and which are well known for their beneficial antioxidant properties. Studies looking at the senolytic properties of a range of flavonoids have found that fisetin is potent [106,107] and roughly twice as potent as quercetin, with an excellent toxicity profile in animal studies [108]. High levels are found in strawberries, apples, persimmons and lower levels in grapes and cucumbers [109]. It has been extensively investigated in vitro and in vivo, where it has wide ranging effects on a number of key pathways involved in cell cycle regulation, apoptosis, the suppression of inflammation, angiogenesis, and metastasis (reviewed in Kashjap at al. [109]). It has been investigated in studies of combined treatment with other anticancer agents to determine whether it may potentiate their effect. These studies are summarised in Table 4.

It is already available for human consumption in low doses as a nutritional supplement as 100 mg capsules where it is marketed to enhance brain health. To put this into context, the average daily consumption of Fisetin is thought to be 0.4 mg per day [109]. In terms of clinical trials, this 100 mg dose has been used in a colorectal cancer chemotherapy study and has been shown to reduce inflammatory markers, although numbers were small and no clinical cancer end points were assessed [121]. There were no safety issues at this dose and the research team felt there was potential for further studies to investigate its adjuvant value.

There is an on-going phase 2 trial (ClinicalTrials.gov Identifier: NCT03675724, AFFIRM_LITE) looking at much higher doses, (20 mg/kg orally for 2 days, so roughly 10- to 15-fold higher than the previous study, with an average 70 kg person getting 1400 mg per day). The study is evaluating the impact of fisetin on a range of biomarkers in older women with frailty syndrome [122]. The initial results are awaited. It is hoped that the AFFIRM LITE study will demonstrate the safety of higher dose Fisetin, to permit clinical trials to assess the potential impact of Fisetin on resilience, age-related dysfunction, and chronic diseases, and as an adjunctive treatment for cancer.

### 12.4. Metformin

As a result of these interactions, there has been recent interest in the use of adjuvant senolytics and senostatics to selectively remove or inhibit senescent cells. For example, the diabetic drug, metformin has senostatic properties and reduces the stimulatory effect of SASP intermediates on prostate cancer cells [123] and is effective in reducing the incidence (chemoprevention) of a range of different cancers [124]. Whether this is due to its senostatic activities or to its impact on metabolism is not yet clear.

### 12.5. Other Agents

A number of other agents with senotherapeutic potential are under investigation. These include HSP90 chaperone inhibitors [125] (such as geldanamycin) and FOXO4 p53 interfering peptide. Whilst trials are in progress with some of these agents, none are yet approved for clinical use. A note of caution is needed in that, whilst some cancers and cancer treatments may react positively to senotherapies, the variation in SASP composition [55] may mean that these findings cannot be extrapolated to other cancers and treatment types.

## 13. Impact of Frailty on Cancer Treatment and Outcomes

There is another way in which senolytics may impact on cancer outcomes—by enhancing resilience and reducing frailty. It is already recognised that long-term survivors of cancer have increased rates of frailty and reduced longevity, some of which are thought to be due to the direct and indirect induction of senescent cells by cancer therapies (chemotherapy and radiotherapy). A trial is currently running to assess the impact of senolytic therapy on stem cell transplant survivors using dasatinib and quercetin in a small number of patients and assessing the impact on frailty [126].

Another important patient group is the elderly with cancer. It is well recognized that treatments such as surgery and chemotherapy have a significant negative impact on physical function, with studies showing an increase in measures of frailty after treatment, which may never recover back to baseline levels. This loss of function is one of the reasons that older patients require longer hospital admission after surgery and sometimes require social care support in the longer term after surgery. This physical dysfunction is therefore a major burden on both the NHS and social care resources and is a research priority for the UK government. If use of senolytic therapies could reduce the frailty phenotype and enhance resilience, this would be a major advance in cancer therapies.

## 14. Conclusions

As can be seen from the above, senescence is a complex process closely related to cell cycle regulatory processes and is integral to human aging and the genesis of many human diseases. It is thought to be a tumour suppressive process, inhibiting both the formation of cancer by blocking transition from premalignant to malignant disease and preventing damaged cells from proliferating after suffering DNA damage or cellular stress, which is one of the key events promoting cancer development. Senescence is also a key effector mechanism of many chemotherapy agents, both directly induced but also via the immune stimulator effects of the SASP. Conversely, it may be a route to tumour resistance and recurrence if the senescent cells reacquire the ability to proliferate when they may become more stem-cell like or via the proinflammatory and apoptosis inhibitory effects of the SASP.

Senotherapies may become a valuable adjunct to cancer therapies both by direct destruction of the chemotherapy-induced senescent cell population within a tumour and by the inhibition of the proinflammatory SASP, which may enhance treatment tolerance by the patient.

Research into the potential of a range of senotherapies is rapidly growing and hopefully new agents will enter clinical practice in the near future.

## Figures and Tables

**Table 1:** Summary of the key characteristics of senescent cells.

Characteristic	Marker
Proliferative arrest	Low expression of Ki-67 or bromodeoxyuridine BrdU
Persistent activation of the DNA damage response	Activation of tumour suppressors, such as p53, p16^INK4A^, cyclins, and cyclin-dependent kinases
Heterochromatic foci (DNA becomes denser than normal)	Senescence-associated heterochromatic foci on DNA staining with DAPI
Cells become flattened and enlarged	Light microscopy changes
Altered metabolism including increased β-galactosidase activity, which is part of carbohydrate metabolism	Measurement of β-galactosidase levels
Senescence-associated secretory phenotype	Interleukins-1, -6, and -8, matrix metalloproteinases, plasminogen activator inhibitor-1

**Table 2:** Table summarising studies of senescence as a prognostic biomarker in various types of cancer.

Model	Cancer Type	Prognostic Significance of Senescence	Reference
Ex vivo human tumours	Breast	Senescence indicates better survival	Althubiti et al. 2014 [29]
Ex vivo human tumours	Hepatocellular carcinoma	Panel of seven senescence associated genes has prognostic significance	Xiang et al. 2019 [46]
Ex vivo human tumours	Squamous head and neck cancer	Senescent cells associated with a non-significant trend to improved prognosis	Schenker et al. 2017 [47]
Ex vivo human tumours	Colorectal cancer	Lower levels of senescence associated with poorer survival	Roxburgh et al. 2013 [48]
Ex vivo human tumours	Lymph node tissue from Hodgkin Lymphoma	High levels of senescence marker expression linked to improved prognosis	Calio et al. 2015 [49]
Ex vivo human tumours	Hepatocellular carcinoma	Low levels of senescence linked to poor prognosis	Mo et al. 2016 [50]
Ex vivo human tumours	Renal cell cancer	Low levels of senescence linked to worse prognosis.	Macher-Goeppinger et al. 2013 [51]

**Table 3:** Table showing studies of Navitoclax in a range of cancer models and trials.

Reference	Model	Drug	Cancer Type	Effect
Tan et al. 2011 [94]	In vitro	Naviticlax+Paclitaxel	Non-small cell lung cancer	More than additive cell killing with combination
Stamelos et al. 2013 [95]	In vitro	Naviticlax+Paclitaxel or Carboplatin	Ovarian cancer	More than additive cell killing with combination
Jeong et al. 2019 [96]	In vitro	Vemurafenib+Navitoclax	Papillary thyroid cancer	Enhanced growth arrest and increase apoptosis with combination
Nakajima et al. 2016 [97]	In vitro	Vorinostat+Navitoclax	Small cell lung cancer	Increased induction of apoptosis with combination
Gonzalez-Gualda et al. 2020 [93]	In vitro and ex vitro	Galactose conjugated Navitoclax+Cisplatin	Lung cancer	Increased cell killing with combination. Reduced platelet toxicity in ex vivo blood
Ackler et al. 2012 [98]	In vivo mouse model	Bendamustine+Navitoclax±Rituximab	Non-Hodgkins lymphoma	Enhanced efficacy in combination
Tolcher et al. 2015 [99]	Phase 1 clinical trial	Irinotecan+Navitoclax	Advanced solid tumours, *n* = 31	6% rate of partial response
Kipps et al. 2015 [100]	Phase 2 clinical trial	Rituximab+Navitoclax	Chronic lymphocytic lymphoma	Combination increase progression free survival and response rates
Rudin et al. 2012 [101]	Phase 2 clinical trial	Single agent navitoclax	Relapsed small cell lung cancer, *n* = 39	23% static disease, 2.6% partial response

**Table 4:** Table summarising studies of fisetin in cancer.

Reference	Model	Drug	Cancer Type	Effect
Li et al., 2018 [110]	In vitro and in vivo mouse xenograft	Fisetin alone	Triple negative breast cancer	Inhibition of proliferation, migration and metastases
Xiao et al., 2018 [111]	In vitro and in vivo mouse xenograft	Fisetin and fisetin micelles	Ovarian cancer	Antiproliferative and proapoptotic effects
Jia et al., 2019 [112]	In vitro and in vivo mouse xenograft	Fisetin alone	Pancreatic cancer	Antiproliferative
Youns et al., 2017 [113]	In vitro	Fisetin alone	Hepatic, colorectal, and pancreatic	Growth arrest and apoptosis
Yan et al., 2018 [114]	In vitro	Fisetin alone	Gastric cancer	Antiproliferative and pro-apoptotic
Yang et al., 2012 [115]	In vitro	Fisetin alone	Breast cancer	Induction of apoptosis
Lin et al., 2015 [116]	In vitro and in vivo	Fisetin and sorafenib	Cervical cancer	Combination superior to either agent alone in anticancer efficacy
Pal et al., 2015 [117]	In vivo mouse model	Fisetin and sorafenib	BRAF mutated melanoma cells	Reduced proliferation, increased apoptosis and reduced metastases in combination
Khan et al., 2019 [118]	In vivo mouse model	Fisetin and 5FU	Colorectal cancer	Reduced incidence of colorectal cancer formation with Fisetin alone and in combination
Zhuo et al., 2015 [119]	In vitro	Fisetin and cisplatin	Lung adenocarcinoma	Increased apoptosis and decreased viability with combination
Touil et al., 2011 [120]	In vivo mouse model	Fisetin and cyclophosphamide	Lung cancer	92% growth inhibition of combination compared to single agent
Farsad-Naemi et al., 2018 [121]	Randomised clinical trial of dietary supplement dose of Fisetin	Oxaliplatin and capecitabine chemotherapy ± Fisetin 100 mg daily for 7 weeks	Colorectal cancer	Reduced levels of inflammatory mediators (IL8, CRP and MMP7) in Fisetin group. Tumour response was not reported
